# Structural Insights into the Molecular Evolution of the Archaeal Exo-β-d-Glucosaminidase

**DOI:** 10.3390/ijms20102460

**Published:** 2019-05-18

**Authors:** Shouhei Mine, Masahiro Watanabe

**Affiliations:** 1 Biomedical Research Institute (BMD), National Institute of Advanced Industrial Science and Technology (AIST), 1-8-31 Midorigaoka, Ikeda, Osaka 563-8577, Japan; 2 Research Institute for Sustainable Chemistry (ISC), AIST, 3-11-32 Kagamiyama, Higashi-Hiroshima, Hiroshima 739-0046, Japan; masa-watanabe@aist.go.jp

**Keywords:** exo-β-d-glucosaminidase, archaea, β-galactosidase, evolution

## Abstract

The archaeal exo-β-d-glucosaminidase (GlmA), a thermostable enzyme belonging to the glycosidase hydrolase (GH) 35 family, hydrolyzes chitosan oligosaccharides into monomer glucosamines. GlmA is a novel enzyme in terms of its primary structure, as it is homologous to both GH35 and GH42 β-galactosidases. The catalytic mechanism of GlmA is not known. Here, we summarize the recent reports on the crystallographic analysis of GlmA. GlmA is a homodimer, with each subunit comprising three distinct domains: a catalytic TIM-barrel domain, an α/β domain, and a β1 domain. Surprisingly, the structure of GlmA presents features common to GH35 and GH42 β-galactosidases, with the domain organization resembling that of GH42 β-galactosidases and the active-site architecture resembling that of GH35 β-galactosidases. Additionally, the GlmA structure also provides critical information about its catalytic mechanism, in particular, on how the enzyme can recognize glucosamine. Finally, we postulate an evolutionary pathway based on the structure of an ancestor GlmA to extant GH35 and GH42 β-galactosidases.

## 1. Introduction

Glucosamine (GlcN) has an array of biological functions and is widely used as a food additive as well as in medicines. GlcN can be enzymatically produced from chitin, which is an abundant bioresource broadly distributed in nature as a major structural component of fungal cell walls, insect exoskeletons, and crustacean shells. Chitin is a β-1,4-linked *N*-acetylglucosamine polysaccharide (GlcNAc)_n_, and its enzymatic conversion to GlcN has become attractive in the chemical industry because it opens a new route for achieving sustainable glucosamine production.

The unique chitin catabolic pathway of hyperthermophilic archaea differs from the known pathways found in other organisms and has been described in *Thermococcus kodakaraensis* KOD1 [1,2,3]. In this pathway, chitin is first degraded into diacetylchitobiose [(GlcNAc)_2_] by chitinase (ChiA) (EC 3.2.1.14), and the acetyl group of the nonreducing side of (GlcNAc)_2_ is deacetylated by a deacetylase (Dac) (EC 3.5.1.105). The resulting product, GlcN-GlcNAc, is subsequently hydrolyzed into GlcN and GlcNAc by an exo-β-d-glucosaminidase (GlmA) (EC 3.2.1.165), followed by further deacetylation of the remaining GlcNAc to GlcN by Dac. These enzymes are thermostable, with an optimal temperature of ~80 °C, which is an important requisite for industrial applications since most industrial processes are conducted under harsh conditions (e.g., high temperature and pressure). Previous determination of the chemical structures of ChiA and Dac provided insights into their catalytic mechanism and adaptation to extremely high temperatures [4,5,6,7,8,9,10]. However, for almost 14 years after the first description of GlmA, its structure has remained unknown.

According to the Carbohydrate-Active Enzymes [CAZy] database, which bases its predictions on the amino acid sequence similarity [11], GlmA belongs to the glycoside hydrolase (GH) 35 family. The other exo-β-d-glucosaminidases found in bacteria and Eukaryota belong to the GH2 [12] and the GH9 [13] families, respectively, and they show little to no sequence similarity to GlmA. Although more than 150 GH families have been classified in the CAZy database, GlmA is almost unique in its sequence, as it presents sequence homology to both GH35 and GH42 β-galactosidases (EC 3.2.1.23) despite its lack of β-galactosidase activity [2,14]. GlmA can hydrolyze various chain lengths of chitooligosaccharides (GlcN_2–6_), cellobiose, and laminaribiose [2]; however, these activities have not been reported for GH35 and GH42 β-galactosidases. Strikingly, the highly conserved motifs around the catalytic residues of these β-galactosidases are not conserved in GlmA [2]. Therefore, it is impossible to predict the key amino acids involved in substrate binding and catalysis of GlmA only from sequence comparisons among these enzymes. 

To address this critical question, we determined the crystal structure of GlmA*_Tk_* (encoded by the TK1754 gene) from *Thermococcus kodakaraensis* KOD1 [15]. The crystal structures of two proteins, which are highly homologous to GlmA*_Tk_*, GlmA*_Ph_* (encoded by the PH0511 gene) [16] and GlmA*_Pf_* (encoded by the PF0363 gene) [14], from the closely related hyperthermophiles *Pyrococcus* species *Pyrococcus horikoshii* and *Pyrococcus furiosus*, respectively, were also determined. The structure of GlmA elucidated the substrate-binding site as well as the substrate selection mechanism. It also revealed that GlmA is a structurally interesting intermediate between GH35 and GH42 β-galactosidases. Here, we review the most recent findings on the structure–function relationship of GlmA and describe the unique structural features that link it to the molecular evolution of glycoside hydrolases.

## 2. Structure and Thermostability of GlmA

**Figure 1:**
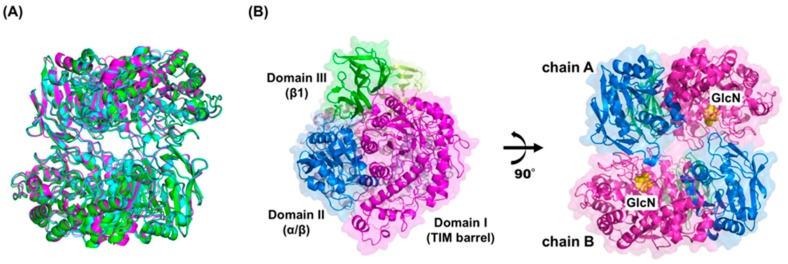
The overall structure of GlmA*_Tk_*. (**A**) The structural superposition of GlmA*_Tk_* (magenta), GlmA*_Ph_* (cyan), and GlmA*_Pf_* (green); (**B**) The dimer structure of GlmA*_Tk_* is presented in two views. GlmA*_Tk_* consists of a homodimer (chains A and B) and comprises three distinct domains (TIM-barrel: magenta, α/β: blue, β1: green). The bound GlcN is represented by yellow van der Waals spheres. The figures were prepared using PyMOL (Schrödinger, LLC, New York, NY, USA).

The structure of GlmA*_Ph_* was deduced using the single-wavelength anomalous dispersion of selenomethionine atoms and refined at 2.60-Å resolution (PDB 5GSL) [15]. The structure of GlmA*_Pf_* and GlcN-bound GlmA*_Tk_* was determined at 1.75-Å resolution (PDB 6JOW, unpublished) and 1.27-Å resolution (PDB 5GSM) [15], respectively, using molecular replacement of the structure of the GlmA*_Ph_* monomer as the search model. The structures of GlmA*_Ph_* and GlmA*_Pf_* showed little variation to that of GlmA*_Tk_,* as reflected in the RMSD values of 0.90 Å for 767 Cα atoms and 0.74 Å for 751 Cα atoms, respectively (Figure 1A). Moreover, both proteins shared high sequence identity with GlmA*_Tk_* (63%) and the active site architecture is fully conserved among these GlmAs (see Section 4.1). These results suggest that general aspects of these proteins, such as the structural features and the catalytic mechanisms, are very likely to be equivalent. GlmA*_Pf_* has been described as a putative β-galactosidase [2,14]. However, structural analysis results indicate that it must be an exo-β-D-glucosaminidase. The highest-resolution complex structure of GlmA*_Tk_* is described throughout this review unless otherwise noted. 

GlcN-bound GlmA*_Tk_* is a homodimer and each monomer (chains A and B) consists of three distinct domains (Figure 1B). Domain I (residues 1–435) is a TIM-barrel structure typical of the GH family. Generally, it contains the catalytic residues [17,18]. As expected, a single molecule of GlcN is located in the bottom of each monomer’s barrel (Figure 1B). Domain II (residues 436–648) is an α/β domain involved in the dimerization process and forms an interface with the TIM-barrel domain of the other monomer. Domain III (residues 649–786) is a β1 domain. There is no structural evidence that this domain is involved in protein activity, but it might contribute to maintaining the overall structural conformation of GlmA*_Tk_*. Indeed, Arg676 from this domain forms hydrogen bonds with His354 and Thr355 from the TIM-barrel domain of the neighboring polypeptide. 

To date, physical and chemical features have been proposed to explain the enhanced protein thermostability [19]. Among them, oligomerization has been considered a form of adaptation to extreme temperatures due to the increase in the number of intermolecular interactions [20,21,22]. For GlmA*_Tk_*, the buried solvent-accessible surface area upon dimer formation is 5530 Å^2^, i.e., 24% of the monomer surface, which is quite large for a dimerization interface. The dimer of GlmA*_Tk_* is held together by numerous interactions at the subunit interface, involving 29 hydrogen bonds and 16 salt bridges per monomer and interactions between at least 36 residues at each monomer interface. Since GlmA*_Tk_* exhibited its highest activity at 80 °C toward GlcN_2_ with an *k*_cat_/*K_m_* value of 0.1 µM^−1^ s^−1^ [2], this stability may be caused by the cumulative effect of these interactions, which may also contribute to the rigidity of the dimer. Recently, the cold-adapted β-galactosidase from *Paracoccus* sp. 32d, *Parβ*DG, a member of the GH2 family, has been reported [23]. Although *Parβ*DG forms a stable dimer, the average B-factor values of *Parβ*DG (37.0 Å^2^) are much higher than those of GlmA*_Tk_* (14.0 Å^2^), indicating that *Parβ*DG has a high degree of flexibility in comparison to that of GlmA*_Tk_*. This result shows that a global conformational rigidity of GlmA*_Tk_* is indeed associated with thermostability.

## 3. Structural Comparison with GlmA Homologous Proteins

**Figure 2:**
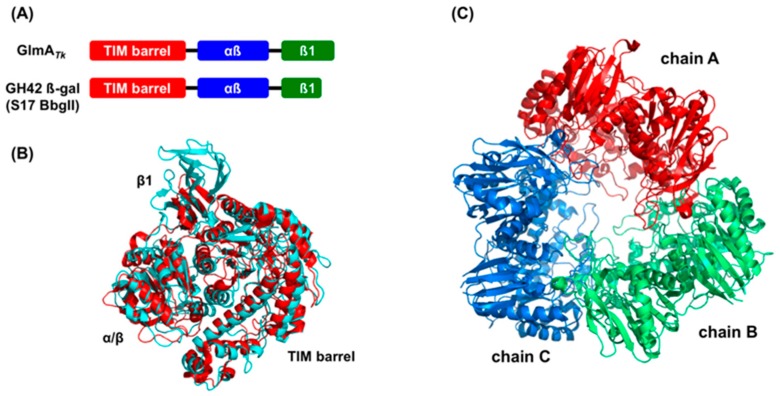
Structural comparison between GlmA*_Tk_* and GH42 β-galactosidases. The comparison was performed with five GH42 β-galactosidases, but, for clarity, only the result of BbgII (PDB 4UZS) is shown in the figure. (**A**) Schematic presentation of the domain organization of GlmA*_Tk_* and BbgII; (**B**) The superimposed models of the monomer structure of GlmA*_Tk_* (cyan) and BbgII (red). The figure was drawn in the same orientation as in the left panel of Figure 1B; (**C**) Trimeric structure of BbgII (chain A: red, chain B: green, chain C: blue).

Bioinformatics analyses using the Dali server [24], which identifies global structural homologs, revealed that the dimer structure of GlmA*_Tk_* does not resemble that of any other protein. However, the three-domain structure of the GlmA*_Tk_* monomer has the same domain order as the GH42 β-galactosidase, although GlmA*_Tk_* is actually a GH35 enzyme. To date, five crystal structures of GH42 β-galactosidases have been reported, including those from *Thermus thermophilus* A4-β-gal (PDB 1KWK) [25], *Bacillus circulans* sp*. alkalophilus* Bca-β-gal (PDB 3TTY) [26], *Geobacillus stearothermophilus* GanB (PDB 4OIF) [27], *Bifidobacterium animalis* BlGal42A (PDB 4UNI) [28], and *Bifidobacterium bifidum* S17 BbgII (PDB 4UZS) [29]. GlmA*_Tk_* and these GH42 β-galactosidases only share 15–17% sequence identity, but their monomer structures could be superimposed with RMSD values of 2.6‒3.0 Å for equivalent Cα atoms, except for 80 additional residues at the C-terminal region of GlmA*_Tk_* (Figure 2A,B). Yet, an important difference is present in the oligomerization state: GH42 β-galactosidases form a homotrimer; thus, their overall structures are quite different from that of GlmA*_Tk_* (Figure 2C).

**Figure 3:**
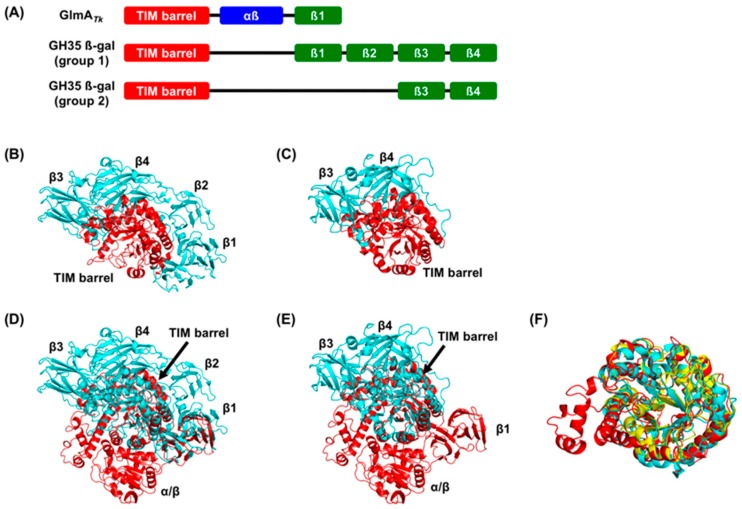
Structural comparison between GlmA*_Tk_* and GH35 β-galactosidases. Seven GH35 β-galactosidases were compared to GlmA*_Tk_*, but, for clarity, only the results of Tri-β-gal (PDB 3OGR) and Hs-β-gal (PDB 3THC) are shown as representatives of Group 1 and 2, respectively. (**A**) Schematic presentation of the domain organization of GlmA*_Tk_*, a Group 1 GH35 β-galactosidase, and Group 2 GH35 β-galactosidase; (**B**) The structure of Tri-β-gal (TIM-barrel: red, β1–4: cyan); (**C**) The structure of Hs-β-gal (TIM-barrel: red, β3 and β4: cyan); (**D**) Superimposed models of GlmA*_Tk_* monomer structure (red) and Tri-β-gal (cyan); orientation, same as that in B; (**E**) Superimposed models of GlmA*_Tk_* monomer structure (red) and Hs-β-gal (cyan); orientation, same as that in C; (**F**) Superimposed models of the TIM-barrel domain (chain A) of GlmA*_Tk_* (red), Tri-β-gal (cyan), and Hs-β-gal (yellow).

Distinctively, a DALI search indicated that the TIM-barrel domain of GlmA*_Tk_* (residues 1–435) bears the highest structural similarity to GH35 β-galactosidases of *Aspergillus oryzae* (Ao-β-gal, PDB 4IUG) [30], *Aspergillus niger* (AnβGal, PDB 5IFP) [31], *Trichoderma reesei* (Tri-β-gal, PDB 3OGR) [32], *Penicillium* sp. (Psp-β-gal, PDB 1XC6) [33], *Homo sapiens* (Hs-β-gal, PDB 3THC) [34], *Bacillus circulans* (Bc-BgaC, PDB 4MAD) [35], and *Streptococcus pneumoniae* (Sp-BgaC, PDB 4E8C) [36]. These GH35 β-galactosidases are roughly divided into two groups: Group 1 comprises the former four β-galactosidases (Ao-β-gal, AnβGal, Tri-β-gal, and Psp-β-gal), and Group 2 is formed by the latter three (Hs-β-gal, Bc-BgaC, and Sp-BgaC). Group 1 β-galactosidases have five domains—a TIM-barrel domain and four β-domains (β1, β2, β3, and β4) (Figure 3A,B). The β-galactosidases in Group 2 show similar domain organization to those of Group 1 but lack the β1 and β2 domains (Figure 3A,C). The domain organization of GH35 β-galactosidases quite differs from that of GlmA*_Tk_* (Figure 3A,D,E). However, the TIM-barrel domains are clearly superimposable, with RMSD values of 1.7‒2.3 Å (Figure 3F). RMSD values for GH42 β-galactosidases were slightly improved (2.3–2.9 Å) when only the TIM-barrel domain was compared, indicating that the TIM-barrel domain of GlmA*_Tk_* is more similar to those of GH35 β-galactosidases than to those of GH42 members. Indeed, a high degree of similarity within the entire catalytic centers was observed between GlmA*_Tk_* and GH35 β-galactosidases, as described below. Collectively, these observations indicate that GlmA*_Tk_*, GH35, and GH42 β-galactosidases are evolutionarily related. 

The TIM-barrel domain and the β1 domain of GlmA*_Tk_* could be superimposed onto those of Group 1 β-galactosidases (Figure 3E). This will be discussed in Section 5.

## 4. GlmA Active Site and Catalytic Mechanism

### 4.1. The Active-Site Architecture of GlmA_Tk_: Comparison with the GH35 β-Galactosidase

We selected the structure of the galactose-bound Hs-β-gal (PDB 3THC) for further comparison with the GlmA*_Tk_* active site because Hs-β-gal is the best-characterized GH35 β-galactosidase to date, both structurally and biochemically [34,37].

**Figure 4:**
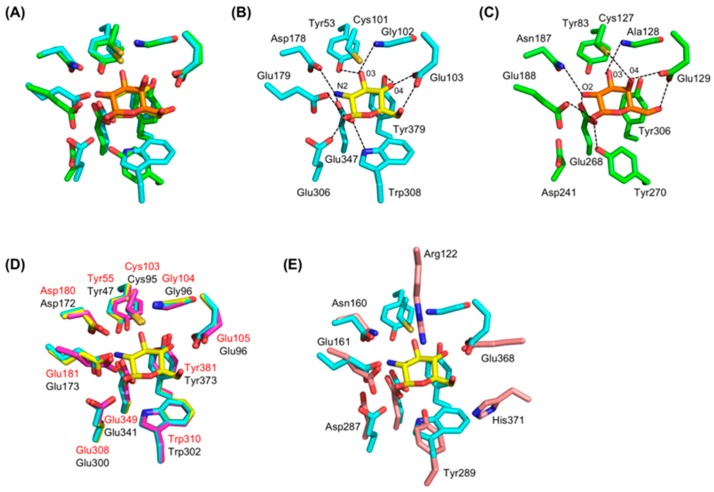
Comparison of the active site residues of GlmA*_Tk_* with those of representative enzymes of the families GH35, GH42, and other GlmAs. (**A**) Superposition of GlmA*_Tk_* (cyan sticks) and Hs-β-gal (green sticks) in complex with GlcN (yellow sticks) and galactose (orange sticks), respectively. Active site residues of GlmA*_Tk_* (**B**) and Hs-β-gal (**C**). Polar interactions are indicated by dashed lines; (**D**) Superposition of GlmA*_Tk_* (cyan sticks with red labels), GlmA*_Ph_* (magenta sticks with black labels), and GlmA*_Pf_* (yellow sticks); (**E**) Superposition of GlmA*_Tk_* (cyan sticks) and BbgII (GH42 β-galactosidase) (pink sticks with black labels). All the figures were drawn in the same orientation as in A.

In GlmA*_Tk_*, a GlcN molecule is bound to each monomer in the chair conformation and it is fixed by making hydrogen bonds with eight residues. Superposition of the TIM-barrel structures of GlmA*_Tk_* and Hs-β-gal yields an RMSD of 1.7 Å over 292 Cα atoms with 32% sequence identity and a strong structural similarity between the −1 subsites of these proteins (Figure 4A). GlcN and galactose, which are different but structurally similar molecules, are located at almost the same position. Interestingly, four of the eight substrate-binding residues in GlmA*_Tk_*, namely, Tyr53, Glu103, Glu179, and Glu347 are present in Hs-β-gal as functionally conserved residues Tyr83, Glu129, Glu188, and Glu268, respectively (Figure 4B,C). These residues form direct hydrogen bonds with the galactose molecule similar to the GlcN-bound GlmA*_Tk_* structure. Gly102 of GlmA*_Tk_* is sterically identical to Ala128 of Hs-β-gal and the main-chain amide of each residue forms a hydrogen bond with the O3 of GlcN/galactose, indicating that this replacement is a conservative substitution. Furthermore, GlmA*_Tk_* Trp308 overlaps well with Hs-β-gal Tyr270 (Figure 4A–C). Tyr270 of Hs-β-gal performs two functions: it maintains the orientation of Glu268 for the hydrogen-bond catalytic reactions and contributes to the formation of the hydrophobic pocket [34]. Trp308 of GlmA*_Tk_* appears to perform the same function through a hydrogen bond to Glu347 (Figure 4B). Remarkably, these residues, which are important for the recognition of GlmA*_Tk_*’s substrate, are structurally conserved in GlmA*_Ph_* and GlmA*_Pf_* (Figure 4D), but they are either composed or located differently in GH42 β-galactosidases (Figure 4E), thus excluding GlmA from the GH42 family classification.

### 4.2. GlmA Catalytic Mechanism Determined through In-Depth Crystallographic Analysis

From the structural comparison, Glu179 and Glu347 of GlmA*_Tk_* are sterically identical to the acid/base Glu188 and the nucleophile Glu268 of Hs-β-gal, respectively (Figure 4A, B, C). GlmA*_Tk_* mutations, E179Q and E347Q, resulted in dramatic activity loss [15], supporting the notion that these residues are involved in protein catalysis. Furthermore, these Glu residues are located in the β4 and β7 strands of the TIM-barrel domain and are separated by 4.8 Å [15]. All proteins in the GH35 family belong to a GH-A clan that comprises enzymes with two conserved catalytic Glu residues in the C-terminals of β4 and β7 [17]. The spatial arrangement of the two GlmA*_Tk_* Glu residues is in entire agreement with the structural features of the GH-A enzymes. Thus, Glu179 and Glu347 act as the acid/base residue and the nucleophilic residue, respectively, and GlmA*_Tk_* cleaves the glycosidic bond through a double-displacement retaining mechanism, like the other GH-A enzymes [17].

**Figure 5:**
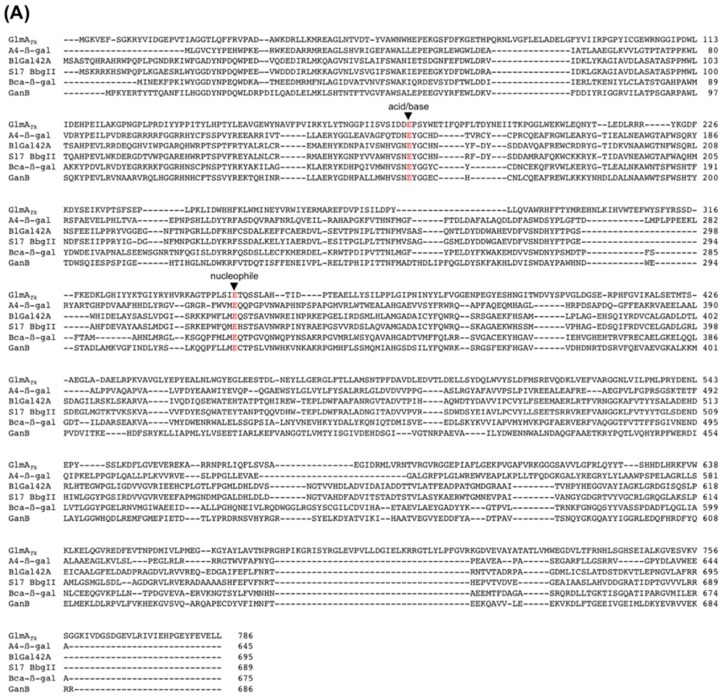

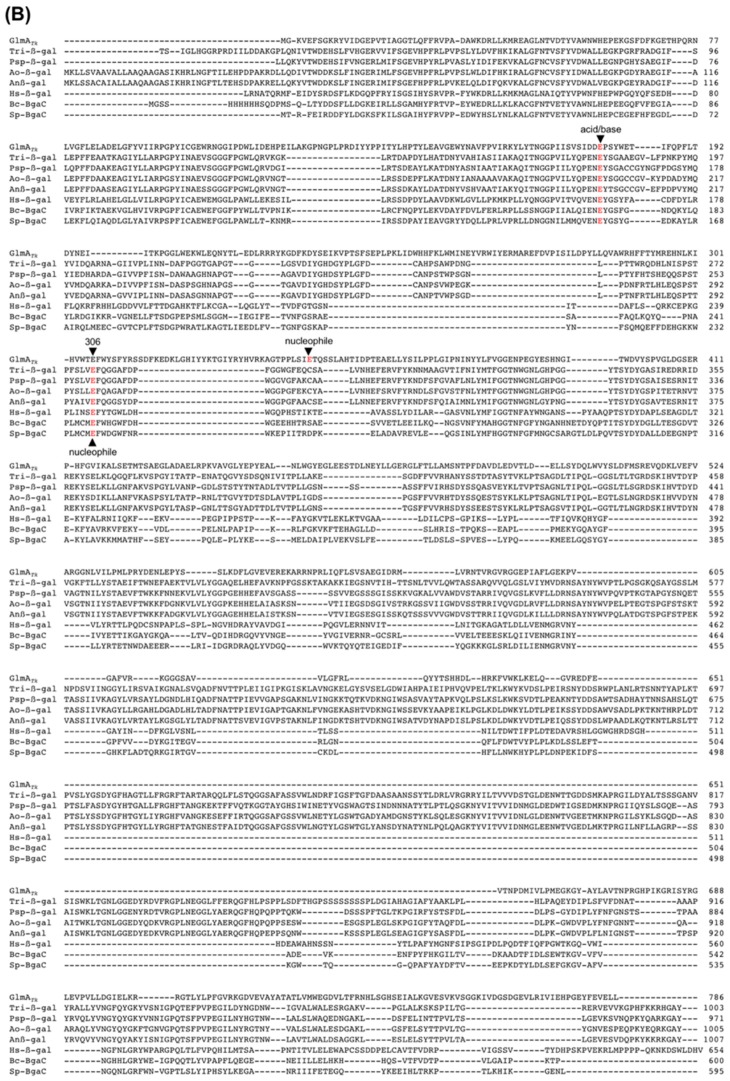
Sequence alignment of the catalytic residues (red) of GH42 β-galactosidases (**A**) and GH35 β-galactosidases (**B**). Alignments were carried out with ClustalW [38].

Despite the absence of sequence identity around the catalytic residues, sequence alignments show that the acid/base Glu179 of GlmA*_Tk_* aligns with the catalytic residues of GH35 and GH42 β-galactosidases (Figure 5A,B). The nucleophile Glu347 of GlmA*_Tk_* also aligns with those of GH42 β-galactosidases (Figure 5A), but not to those of GH35 β-galactosidases (Figure 5B). Instead of Glu347, GlmA*_Tk_* Glu306 locates at the position that corresponds to the nucleophilic residue of GH35 β-galactosidases (Figure 5B). Glu306 forms a hydrogen bond with GlcN O1 (Figure 4B) and contributes to the protein’s catalytic activity [15]. However, in Hs-β-gal, this position is structurally occupied by an Asp residue (Asp241), which is found in almost all GH35 β-galactosidases [30,31,32,33,34,36]. Asp241 cannot form a hydrogen bond with galactose because of its side chain orientation (Figure 4C) and, for this reason, should not be involved in the catalytic reaction. These results strongly suggest that a prediction of GlmA*_Tk_*’s catalytic residues from sequence comparisons is not reliable and that the catalytic mechanisms could only be determined through in-depth crystallographic analysis. 

### 4.3. The Role of Asp178

Several unique structural features of GlmA*_Tk_* can provide insights into its substrate recognition mechanisms. The most important substrate-recognizing residue is Asp178, which precedes the acid/base Glu179 residue. The Asp–Glu motif replaces the conserved Asn–Glu motif (equivalent to the Asn187–Glu188 motif in Hs-β-gal) in all GH35 and GH42 β-galactosidases. Hs-β-gal Asn187 forms a hydrogen bond with the C2–OH of galactose (Figure 4C), while GlmA*_Tk_* Asp178 forms a hydrogen bond with the C2–NH_2_ of GlcN (Figure 4B). The p*K*_a_ values of the Asp178 carboxyl group and of the GlcN N2 group are approximately 3.7 and 7.4 [39], respectively. Therefore, at pH 6.0, at which GlmA*_Tk_* activity is the highest [2], negatively charged Asp178 could interact with the protonated NH_3_^+^ form of N2. To confirm this hypothesis, a D178N mutant was created and experimental results showed that it was inactive [15], implying that the charge–charge interaction is a major factor for the GlmA*_Tk_* recognition of GlcN. This assumption is supported by a previous observation that GlmA*_Tk_* has very weak β-glucosidase activity [2]. Glucose differs from GlcN only at the C2 substituent, which is C2–OH, and the loss of a charged interaction between Asp178 and the C2–OH of glucose should result in fundamental loss of β-glucosidase activity. On the other hand, GlcNAc differs from GlcN only at the C2 substituent, which contains a large acetoamide group. This group would sterically clash with Asp178, in accordance with GlmA*_Tk_* being completely unable to hydrolyze (GlcNAc)_2_ [2]. These results suggest that Asp178 is a key residue because of its ability to discriminate between substrates.

CsxA from *Amycolatopsis orientalis*, a member of the GH2 family, is the only other exo-β-d-glucosaminidase with a known structure [40,41]. In CsxA, Glu394 binds to GlcN C2–NH_2_ by means of a charged interaction [40], similar to that of GlmA*_Tk_*. However, Glu394 is distantly located from the Asp469 acid/base residue in the CsxA sequence, and the residue that precedes that acid/base residue is Ser468. Therefore, the use of an Asp–Glu motif to discriminate among substrates is only found in GlmA glycoside hydrolases characterized thus far.

### 4.4. Residue Conservation during Evolution

The other remarkable GlmA*_Tk_* conserved residues are Cys101 and Tyr379, which could be superimposed onto Hs-β-gal Cys127 and Tyr306, respectively (Figure 4A–C). These two residues are conserved in almost all GH35 β-galactosidases [34,36]. GlcN and galactose differ in their chirality of O4, which is equatorial in GlcN and axial in galactose. GlmA*_Tk_* Tyr379 forms a hydrogen bond (2.8 Å) with the equatorial O4 of GlcN (Figure 4B) and provides an aromatic stacking interaction with GlcN through a hydrophobic platform for the C4 side. Hs-β-gal Tyr306 also serves as a hydrophobic stacking platform to accommodate galactose. However, it cannot provide a hydrogen bond to the axial O4 of galactose because it is very distant from it (4.6 Å) (Figure 4C). Instead, Hs-β-gal Cys127 forms a hydrogen bond (3.3 Å) with the axial O4 of galactose via its thiol group (Figure 4C). Likewise, GlmA*_Tk_* Cys101 is present as Hs-β-gal Cys127 counterpart, but its location is too far (4.8 Å) to form a hydrogen bond with the equatorial O4 of GlcN (Figure 4B). In brief, GlmA*_Tk_* and GH35 β-galactosidases possess residues that could form hydrogen bonds with axial and equatorial O4 forms in the glycosidic substrate. The presence of Cys and Tyr residues is regarded as a remnant of evolution. As far as we know, such residues have never been seen in different functional glycoside hydrolases in the course of evolution, and they constitute a compelling link to the molecular evolution of these enzymes. 

### 4.5. GlmA Dimer Structure Influences Substrate Specificity

**Figure 6:**
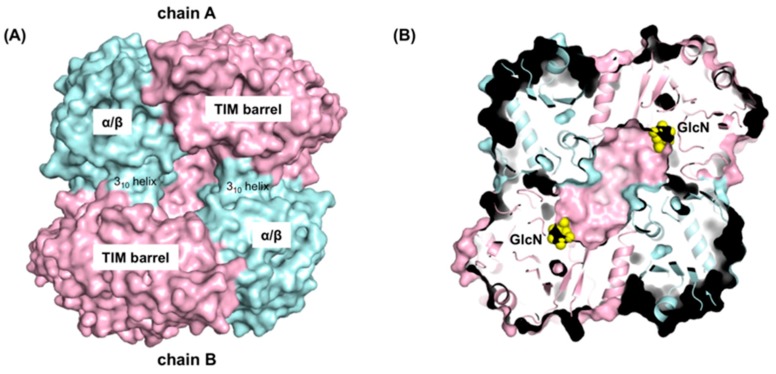
The dimerization interface and the deep active site pocket of GlmA*_Tk_*. (**A**) Surface representation of the GlmA*_Tk_* dimer (TIM-barrel: light pink, α/β: cyan). The figure was drawn from the same orientation as in the right panel of Figure 1; (**B**) Section drawing of the GlmA*_Tk_* dimer containing GlcN molecules (yellow) in the active site. The figure was drawn in the same orientation as in A.

As shown in Figure 1B and Figure 6B, the active sites of GlmA*_Tk_* are located within a deep pocket that intrudes into the core of the TIM-barrel domain of each monomer. Such active sites may act independently and their entrances, which are the only means of passage for substrates and products, are created by a reciprocal donation of each monomer. That is, the 3_10_-helix of the α/β-domain of chain A interacts with the TIM-barrel domain of chain B, narrowing the active site entrance. As a result, the distance of subsite -1 from the active site entrance is approximately 20 Å (Figure 6A,B), which may contribute to a size selection. Indeed, GlmA*_Tk_* showed higher activity against GlcN_2_ (approximately 12 Å in length) and its activity decreased in proportion to the length of the chitooligosaccharide chain [2]. These results suggest that dimer formation enables GlmA*_Tk_* to form an active site with an appropriate shape for binding smaller substrates. 

## 5. Molecular Evolution of GlmAs and β-Galactosidases

The crystal structure of GH35 GlmA*_Tk_* shows structural similarities to both GH35 and GH42 β-galactosidases. Briefly, the monomeric structure of GlmA*_Tk_*, which comprises the TIM-barrel domain, the α/β domain, and the β1 domain, is similar to that of GH42 β-galactosidases, whereas in the TIM-barrel domain, the key amino acids involved in substrate binding and catalysis at subsite -1 are highly conserved between GlmA*_Tk_* and GH35 β-galactosidases. As previously stated, the sequence of GlmA*_Tk_* bears homology to sections of GH35 and GH42 β-galactosidases [2]. Therefore, these β-galactosidases may have evolved from GlmA*_Tk_* via gene duplication, truncation, or domain insertion.

GlmA*_Tk_* and GH42 β-galactosidases are active as a dimer and a trimer, respectively. They have a cleft-type active site in their monomeric forms, which is suitable for binding to long-chain polysaccharides. Despite having different oligomerization states, both enzymes change the active site from the cleft to the pocket-type upon oligomerization to better accommodate smaller substrates. Moreover, Juers et al. reported other features that reduce the size of the active site [42]: a lengthening loop at the end of the TIM-barrel domain that partially fills in the active site cleft; and the addition of extra domains on either side of the active site cleft. During molecular evolution, lengthening loops would be more efficient than oligomerization or the addition of domains, but it seems that evolutionary selection gives priority to function over efficiency [42]. For GH42 β-galactosidases, trimer formation is essential to exhibit high enzymatic activity as well as to ensure size-based substrate specificity [25,27,28]. Thus, the use of GlmA*_Tk_*’s monomer structure frameworks (i.e., domain organization) might be necessary for fulfilling its functions via trimer formation. However, the substrate-binding residues of GlmA*_Tk_* are not well conserved in GH42 β-galactosidases (Figure 4E) and the underlying evolutionary selection pressure that led to this diversity in the active site remains unknown. 

In GH35 β-galactosidases, the original substrate-binding residues of GlmA*_Tk_* are highly conserved, and those with some conservative substitutions (e.g., Trp308 of GlmA*_Tk_* is substituted in Hs-β-gal by Tyr270) and the catalytic machinery were retained, whereas the reaction specificity has evolved toward β-galactosidase activity. In other words, the GH35 β-galactosidase could have evolved from ancestral GlmA*_Tk_* to be able to recognize galactose through a subtle change of residues around subsite −1. Indeed, a single residue, GlmA*_Tk_* Asp178, replaces the conserved Asn residue in the GH35 β-galactosidase and plays an essential role in the recognition of GlcN. In addition, GlmA*_Tk_* Cys101 and Tyr379, which are well conserved and similarly spatially located in the GH35 β-galactosidase, have the potential for forming hydrogen bonds with either the axial (galactose) or equatorial (GlcN) forms of O4 in the glycosidic substrate, respectively. This further supports the proposed evolutionary approach. The underlying mechanisms could be driven by constraints in the availability of different substrates in the organism’s habitat or in the ability to survive [43,44]. Therefore, the change in GlmA*_Tk_* substrate specificity might have developed under the positive constraint of galactose presence.

**Figure 7:**
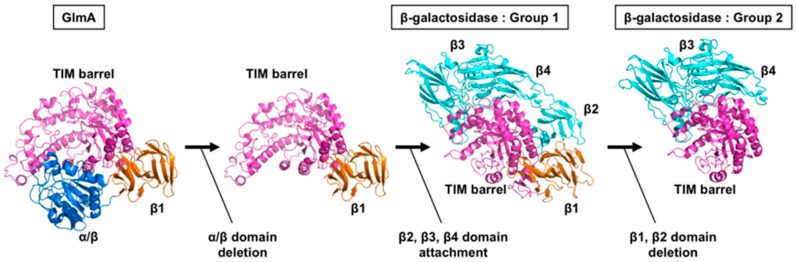
Hypothetical evolutionary pathway from GlmA to GH35 β-galactosidases. TIM-barrel domain, α/β: domain, β1 domain, and β2–β4 domains are colored magenta, blue, orange, and cyan, respectively.

Although the GlmA*_Tk_* substrate-binding scaffold is almost entirely retained in GH35 β-galactosidases, the domain organization is different (Figure 3A). It has been suggested that evolutionary pathways can be tracked at the structural level [25,45,46]. Moreover, Matthews et al. proposed the evolutionary path of β-galactosidase from *Escherichia coli* (Ec-β-gal), an enzyme that belongs to the GH2 family and is the best studied β-galactosidase [42,47]. Ec-β-gal is a homotetramer, its monomer structure can be divided into five domains, and is built around the TIM-barrel with the remaining four domains similar to the Group 1 GH35 β-galactosidase. First, Ec-β-gal may have evolved from a much smaller enzyme, such as a single TIM-barrel domain, that cleaves long polysaccharides. Second, during the modulation of the substrate specificity, additional domains may have been added. Based on this scenario, we speculate that an early GH35 β-galactosidase ancestor with a structure similar to that of Group 1 β-galactosidases (Ao-β-gal, AnβGal, Tri-β-gal, and Psp-β-gal) may have first arisen from GlmA*_Tk_* via deletion of the α/β domain, which could accommodate extended substrates (Figure 7). This hypothesis is supported by the observation that the TIM-barrel domain and the β1 domain of GlmA*_Tk_* could be superimposed onto those of Group 1 β-galactosidases (Figure 3E). Subsequently, addition of the β2, β3, and β4 domains could then have conferred the substrate specificity on the enzymes. The extended loop from the β3 domain especially not only contains the substrate specificity determinant residue, but also constitutes a part of the active site pocket [36]. Although the functions of the β1, β2, and β4 domains remain unknown, they seem to stabilize the complete structure of Group 1 β-galactosidases. The final step—deletion of the β1 and β2 domains—could have led to the appearance of Group 2 β-galactosidases (Hs-β-gal, Bc-BgaC, and Sp-BgaC) (Figure 7). Interestingly, Group 2 β-galactosidases form dimers, whereas other β-galactosidases function as monomeric enzymes, suggesting that a deletion of the β1 and β2 domains may be needed for dimer formation. In other words, Group 2 β-galactosidases may form a dimer to compensate the instability caused by the deletion of the β1 and β2 domains.

## 6. Conclusions

GlmA*_Tk_*, GH35, and GH42 β-galactosidases belong to the same GH-A clan. A clan is a group of families that show significant similarities in the tertiary structure as well as conservation of catalytic residues and mechanisms, and its members are therefore considered to have common ancestry [11]. Accordingly, our findings presented here strongly suggest that GlmA*_Tk_* is a common ancestor of both GH35 and GH42 β-galactosidases.
